# Is Ultrasound a Reliable and Reproducible Method for Assessing Adnexal Masses in Pregnancy? A Systematic Review

**DOI:** 10.7759/cureus.19079

**Published:** 2021-10-27

**Authors:** Jonathan E Gaughran, Osama Naji, Mohammed Q Al Sabbagh, Ahmad Sayasneh

**Affiliations:** 1 Women’s Health, Guy’s and St Thomas’ NHS Foundation Trust, London, GBR; 2 Gynaecology, Guy’s and St Thomas’ NHS Foundation Trust, London, GBR; 3 Department of Neurology, University of Kansas Medical Center, Kansas City, USA; 4 School of Life Course Sciences, Faculty of Life Sciences and Medicine, King’s College London, London, GBR; 5 Gynaecological Oncology, Guy’s and St Thomas’ NHS Foundation Trust, London, GBR

**Keywords:** diagnostics, ultrasonography, pregnancy, adnexal mass, ovarian neoplasm

## Abstract

In this study, we aimed to systematicallyreview the current evidence regarding the diagnostic accuracy of ultrasound in assessing adnexal masses in pregnancy. The Cochrane Register of Controlled Trials, PubMed, and EMBASE databases were searched for all types of clinical studies that utilised ultrasound for the diagnosis of adnexal masses in pregnancy. Only studies that used outcome measures of either histological diagnosis or significant regression of the adnexal mass on imaging follow-up were included. The quality of each study was assessed for risk of bias. The diagnostic performance of ultrasound in each study type was calculated, along with the pooled diagnostic performance of ultrasound in differentiating benign from malignant masses. The initial search yielded 4,915 articles, of which 2,547 qualified for abstract screening. A total of 83 articles were included in this review, including one prospective cohort study, six retrospective observational studies, seven case series, and 69 case reports. In the included studies, the total number of adnexal masses was 559. The mean patient age was 29.2 years (95% confidence interval [CI]: 28.7-29.7), with a mean gestational age at diagnosis of 13.8 weeks (95% CI: 13.2-14.4). The mean quality assessment score was 75%. The International Ovarian Tumour Analysis Simple Rules were used in two articles, whereas subjective impression was used in the remaining 81 articles. The most frequently diagnosed mass was a simple or physiological cyst (35%). The prevalence of malignancy in the entire sample was 46/559 (8%; 95% CI: 34-61%). The overall pooled sensitivity, specificity, positive likelihood ratio, and negative likelihood ratio of ultrasound in detecting ovarian malignancy were 64% (95% CI: 30-88%), 88% (95% CI: 64-97%), 5.6 (95% CI: 1.2-25.4), and 0.4 (95% CI: 0.15-1), respectively. In conclusion, currently, there is a lack of high-quality prospective studies to guide the management of adnexal masses in pregnancy. Ultrasound appears to have an adequate accuracy in differentiating benign from malignant masses; however, more research is required to assess the role of ultrasound models, rules, and subjective assessment in pregnancy compared to non-pregnant women.

## Introduction and background

Adnexal masses in pregnancy are common, with an incidence ranging from 0.19% to 8.8% [1]. The utilisation of ultrasound for fetal assessment leads to incidental findings of adnexal masses, the majority of which are physiological [2]. With improvement in ultrasound technology, detection rates have increased. Malignancy is rare in this cohort, and surgery during pregnancy is associated with adverse maternal and fetal outcomes [1,3]. As such, conservative management is favoured when possible. However, pregnancy poses challenges for the ultrasound practitioner in determining the nature of adnexal masses. Due to anatomical adaptations, the transvaginal approach is of limited value in late gestation. Moreover, adaptations to ovarian blood flow may alter Doppler findings, and morphological changes in endometriomas can mimic malignancy [4,5]. Ultrasound rules and models that improve the diagnostic accuracy of adnexal masses such as the International Ovarian Tumour Analysis (IOTA) ‘Simple Rules’ have not been ratified in pregnancy. Limited data on magnetic resonance imaging (MRI) suggest that it is safe in pregnancy [2]. However, experience in interpreting these images is limited. The inability to administer gadolinium due to its teratogenicity is a limitation of MRI, and movement artefacts from the fetus reduce image quality [6,7]. Compared to MRI, ultrasound is cheaper and readily accessible, which makes it a preferred modality. The objective of this study was to systematically review the available data on the diagnostic performance of ultrasound in differentiating benign from malignant masses in pregnancy. Secondary objectives were to assess the characteristics of adnexal masses in pregnancy reported in the literature and to assess if any ultrasound rules or models are being used to characterise such masses.

Methodology

Search Strategy

Articles were identified by conducting a literature search using PubMed, EMBASE, and The Cochrane Register of Controlled Trials from January 2000 to January 2021. The title, abstract, and MeSH terms were searched for all combinations of words for adnexa (ovary, ovarian, Fallopian tube, tubal, broad ligament, parametrial, parametrium); adnexal mass (cyst, tumour, neoplasm, malignancy, borderline tumour, adenoma, dermoid, teratoma, corpus luteum, corpora, endometrioma); imaging (ultrasound, transvaginal, transabdominal, computed tomography, CT, magnetic resonance imaging, MRI, MR); and pregnancy (pregnant, gravid, antenatal, gestational). This electronic search strategy is elaborated in Appendix 1, 2. The reference lists of included studies were cross-referenced to identify articles that were not captured by our search.

Inclusion Criteria

All studies that identified adnexal masses in pregnancy and used outcome measures of histopathological diagnosis were included. If the histological diagnosis was not available, sufficient follow-up imaging with satisfying evidence to the examiner of benignity, such as significant reduction in size (>50%), complete resolution of the mass, or disappearance of any suspicious feature, was required as criteria for the study to be included in the systematic review. Only full papers published in peer-reviewed journals in the English language were assessed. Given the limited number of randomised controlled trials or large cohort studies, no additional methodological filters were applied. Studies were selected in a two-stage process by two authors (JG and ON). First, eligibility was assessed based on the title and abstract. Second, the full article was examined to determine inclusion suitability. If there was disagreement, a co-author (AS) was consulted for the final decision.

Data Extraction

Data extraction was performed by one author (JG). The following information was recorded (when available): patient age; ethnicity; gestation at diagnosis; presence or lack of symptoms; whether ultrasound was performed transvaginally or transabdominally; and whether ultrasound assessment tools were used (e.g. pattern recognition or IOTA Simple Rules). Decisions to manage conservatively or surgically were noted. In addition, whether resolution/reduction in the size of the mass occurred, or whether histopathology was in keeping with the ultrasound diagnosis was also noted. The utilisation of MRI and its findings were also recorded.

Presentation and Quality Assessment of Data

The Preferred Reporting Items for Systematic Reviews and Meta‐Analyses (PRISMA) statement was used for reporting the methods, results, and discussion of this review [8]. The Strengthening the Reporting of Observational Studies in Epidemiology (STROBE) statement was used to assess the quality of the prospective and retrospective cohort studies, whereas the Joanna Briggs Institute (JBI) checklist was used for the case reports and case series (Appendix 3-5) [9,10]. A total of 34 points can be awarded to studies using the STROBE list, 16 for the JBI checklist for case reports, and 20 for the JBI checklist for case series. This quality assessment was performed independently by two authors (JG and ON), followed by consultation with a co-author (AS) in cases of disagreement. The full STROBE and JBI checklists are provided in Appendix 3-5.

Statistical Analysis

Due to similarities in study design, the case reports and case series are presented collectively. Due to differences in design and methodology, the prospective and retrospective observational studies are presented individually. The 2 × 2 contingency tables were constructed using MedCalc to calculate the sensitivity, specificity, positive predictive value (PPV), negative predictive value (NPV), and accuracy (with 95% confidence intervals [CIs]) of the performance of ultrasound in each study type [11]. The agreement rate between ultrasound and MRI was also calculated. For pooled calculations, any masses diagnosed on ultrasound as ‘complex’ or ‘unclassifiable’ were excluded from the 2 × 2 contingency tables. Because of the low estimate weight, case reports and case series were not included in the meta-analysis pooling. To pool our data with balanced weighing, case reports and case series were excluded. Data regarding ultrasound performance were extracted from seven studies. A random-effects model was used to determine pooled sensitivity, specificity, positive likelihood ratio (LR+), and negative likelihood ratio (LR−). To characterise the clinical utility of a test and to estimate the post-test probability of disease, LR+ and LR− were used. An LR value of 0.2-5.0 was proposed to provide weak evidence for ruling out or confirming the disease; an LR value of 5.0-10.0 and 0.1-0.2 provided moderate evidence, and an LR value of >10 or <0.1 provided strong evidence to either confirm or rule out the disease [12]. Summary receiver-operating characteristics (sROC) curves were plotted to illustrate the relationship between sensitivity and specificity.

All analyses were performed using Meta-Analytical Integration of Diagnostic Accuracy Studies (MIDAS) and METANDI commands in STATA version 14.0 for Windows (Stata Corp., College Station, TX, USA). A p-value of <0.05 was considered statistically significant.

## Review

Results

Literature Identified

**Figure 1:**
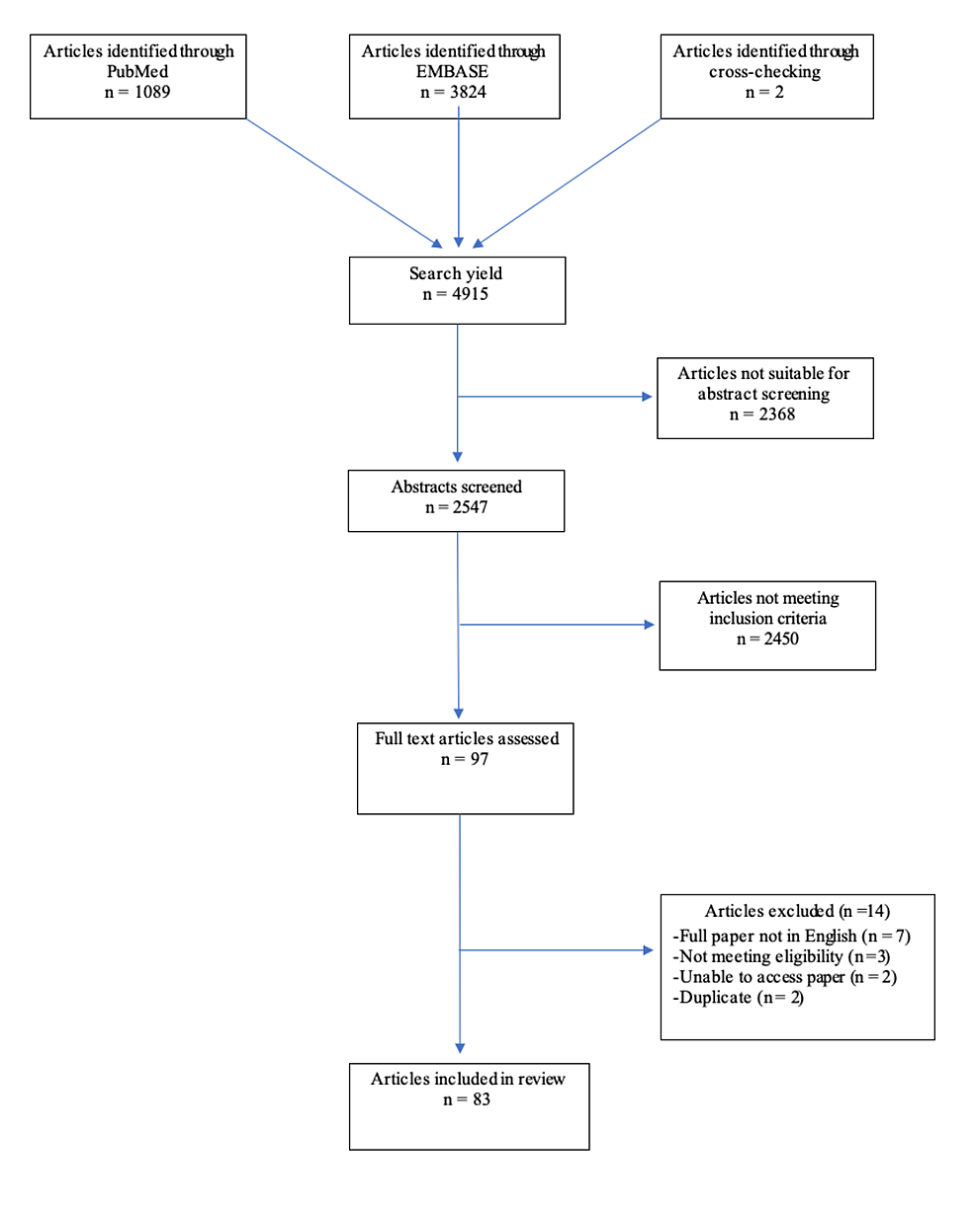
PRISMA flow diagram of the literature search. PRISMA: Preferred Reporting Items for Systematic Reviews and Meta-Analyses

The electronic search of the three databases yielded 4,913 articles. Cross-checking of references identified two additional papers. Of these 4,915 studies, 2,547 met the eligibility criteria for abstract screening. Initially, 97 were deemed to meet the inclusion criteria; however, 14 were subsequently excluded for the following reasons: full paper not available in English (seven), did not meet eligibility criteria (three), inability to access the article (two), duplicate articles (two). This is depicted in Figure 1. Finally, 83 articles were included in this review, comprising one prospective cohort study, six retrospective observational studies, seven case series, and 69 case reports. There were 559 adnexal masses. Case reports and case series had a combined adnexal mass count of 92. The prevalence of malignancy in the entire sample was 8% (46/559) (95% CI: 34-61%).

Quality Assessment of Studies

The quality assessment scores ranged from 50% to 100%, with a mean of 75%. Quality assessment scores along with four of the most relevant assessment criteria are shown in Tables 1-3.

**Table 1:** Quality assessment of included case series using the JBI checklist with the four most relevant criteria and percentage scoring. JBI: Joanna Briggs Institute; Y: yes; N: no

Study	Design	Objectives	Descriptive data	Key results	Interpretation	%
Eichenberger-Gautschi et al., 2018 [14]	Retrospective cohort	Y	Y	Y	Y	83
Pateman et al., 2014 [13]	Retrospective cohort	Y	N	Y	Y	71
Bailleux et al., 2015 [4]	Retrospective cohort	Y	Y	N	Y	70
Surampudi et al., 2015 [15]	Retrospective cohort	Y	Y	N	Y	70
Mascilini et al., 2014 [16]	Retrospective cohort	N	N	N	Y	67
Zanetta et al., 2003 [17]	Prospective cohort	Y	N	N	Y	63
Dobashi et al, 2012 [18]	Retrospective cohort	Y	Y	N	N	59

**Table 2:** Quality assessment of included case series using the JBI checklist with the four most relevant criteria and percentage scoring. JBI: Joanna Briggs Institute; Y: yes; N: no

Study	Clear inclusion criteria	Valid method for identification of condition	Standard and reliable method for all	Clear clinical information	Outcomes clearly reported	%
Barbieri et al., 2009 [19]	Y	Y	Y	N	Y	89
Machida et al., 2008 [20]	Y	Y	Y	Y	Y	78
Xie et al., 2015 [21]	Y	Unclear	Y	Y	Y	72
Hasiakos et al., 2008 [22]	N	Unclear	Y	Y	Y	56
Suzuki et al., 2004 [23]	N	Unclear	Y	Y	Y	56
Yoshida et al., 2008 [24]	Y	Unclear	Y	Y	Y	56
Sammour et al., 2005 [25]	N	Y	Y	Y	Y	50

**Table 3:** Quality assessment of included case reports using the JBI checklist with the four most relevant criteria and percentage scoring. JBI: Joanna Briggs Institute; Y: yes; N: no

Study	Clinical history	Diagnostic test	Intervention	Takeaway lessons	%
al-Harbi et al., 1998 [26]	Y	Y	Y	Y	100
Detti et al., 2011 [27]	Y	Y	Y	Y	100
Grigoriadis et al., 2014 [28]	Y	Y	Y	Y	100
Jabeen et al., 2017 [29]	Y	Y	Y	Y	100
Taylor et al., 2015 [30]	Y	Y	Y	Y	100
Abe et al., 2011 [31]	Y	Y	Y	Y	87
Amaratunga et al., 2018 [32]	Y	Y	Y	Y	87
Amoah et al., 2011 [33]	Y	Y	Y	Y	87
Baksu et al., 2004 [34]	Y	Y	Y	Y	87
Cacciottola et al., 2016 [35]	Y	Y	Y	Y	87
Casanova et al., 2013 [36]	Y	Y	Y	Y	87
Cochrane et al., 2020 [37]	Y	Y	Y	Y	87
Chen et al., 2017 [38]	Y	Y	Y	Y	87
Chaudhry et al., 2013 [39]	Y	Y	Y	Y	87
Devlin et al., 2020 [40]	Y	Y	Y	Y	87
Eftekhar et al., 2005 [41]	Y	Y	Y	Y	87
Edell et al., 2018 [42]	Y	Y	Y	Y	87
El-Agwany, 2014 [43]	Y	Y	Y	Y	87
Fruscella et al., 2004 [44]	Y	Y	Y	Y	87
Gaurilcikas et al., 2020 [45]	Y	Y	Y	Y	87
Gaspar-Oishi et al., 2012 [46]	Y	Y	Y	Y	87
Hitzerd et al., 2006 [47]	Y	Y	Y	Y	87
Ibraheim et al., 2005 [48]	Y	Y	Y	Y	87
Inamdar and Loo, 2019 [49]	Y	Y	Y	Y	87
Kole et al., 2016 [50]	Y	Y	Y	Y	87
Kolluru et al., 2009 [51]	Y	Y	Y	Y	87
Lager et al., 2018 [52]	Y	Y	Y	Y	87
Luh et al., 2019 [53]	Y	Y	Y	Y	87
McCormick et al., 2009 [54]	Y	Y	Y	Y	87
Nakai et al., 2015 [55]	Y	Y	Y	Y	87
Nguyen et al., 2019 [56]	Y	Y	Y	Y	87
Onodera et al., 2008[57]	Y	Y	Y	Y	87
Ozdegirmenci et al., 2007 [58]	Y	Y	Y	Y	87
Pasternak et al., 2014 [59]	Y	Y	Y	Y	87
Pepe et al., 2019 [60]	Y	Y	Y	Y	87
Prefumo et al., 2009 [61]	Y	Y	Y	Y	87
Izza Rozalli et al., 2015 [62]	Y	Y	Y	Y	87
Sorrentino et al., 2020 [63]	Y	Y	Y	Y	87
Soule et al., 2020 [64]	Y	Y	Y	Y	87
Sanaullah and Trehan, 2011 [65]	Y	Y	Y	Y	87
Sanaullah and Trehan, 2009 [66]	Y	Y	Y	Y	87
Sayasneh, 2012 [5]	Y	Y	Y	Y	87
Tazegül et al., 2013 [67]	Y	Y	Y	Y	87
Uccella et al., 2020 [68]	Y	Y	Y	Y	87
Teoh et al., 2003 [69]	Y	Y	Y	Y	81
Wang et al., 2019 [70]	Y	Y	Y	Y	81
Chaverri et al., 2019 [71]	Y	Y	Y	Y	72
Malak and Klam, 2015 [72]	Y	Y	Y	Y	72
Mavromatidis et al., 2010[73]	Y	Y	Y	Y	67
Tannus et al., 2009 [74]	Y	Y	Y	Y	67
Ziruma et al., 2019 [75]	Y	Y	Y	Y	67
Husz et al., 2018 [76]	Y	Y	Y	Y	66
Rao et al., 2018 [77]	Y	Y	Y	Y	66
Tahmasebi et al., 2019 [78]	Y	Y	Y	Y	66
Yen et al., 2000 [79]	Y	Y	Y	Unclear	61
Co et al., 2014 [80]	Y	Y	Y	Y	56
Duru Coteli et al., 2018 [81]	Y	Y	Y	Y	56
Felemban et al., 2019 [82]	Y	Y	Y	Y	56
Hummeida et al., 2015 [83]	Y	Y	Y	Y	56
Khurana et al., 2017 [84]	Y	Y	Y	Y	56
Parveen et al., 2007 [85]	Y	Y	Y	Y	56
Perillo et al., 2020 [86]	Y	Y	Y	Y	56
Zhang et al., 2014 [87]	Y	Y	Y	Y	56
Donnadieu et al., 2006 [88]	N	Y	Y	Y	50
Golasa et al., 2019 [89]	Y	Y	N	Y	50
Kalmantis et al., 2011 [90]	Y	Y	Y	Y	50
Poder et al., 2008 [91]	Y	Y	Y	Y	50
Takeuchi et al., 2019 [92]	N	Y	Y	Y	50

Demographics

The studies were conducted in high, middle, and low-income countries in a mix of tertiary referral units and district hospitals. The mean age of patients was 29.2 (95% CI: 28.7-29.7) years. The vast majority of studies did not define the ethnicity of the patients. The mean gestational age (in weeks) at the time of ultrasound diagnosis was 13.8 ± 7.4 (95% CI: 13.2-14.4), which ranged between 5 and 35. Patients reported symptoms in 33% (95% CI: 26.5-33.5%) of cases. In 81 of the 83 studies, the modality of ultrasound scan used was transabdominal in 52/83 (63%), transvaginal in 22/83 (27%), and a combination of the two in 7/83 (8%). The prospective trial by Zanetta et al. was the only study that assessed the reliability of ultrasound in the diagnosis of adnexal masses in pregnancy [17]. Two studies used IOTA Simple Rules, and only one study stated the level or profession of the ultrasound practitioner [7,17]. In 93 of the 559 masses (17%), an ultrasound impression of either ‘malignant’, ‘complex’, or ‘unclassifiable’ was provided. Table 4 summarises the sensitivity, specificity, PPV, NPV, and accuracy of ultrasound in differentiating benign from malignant masses. Due to the varying methodology of the articles, not all values could be calculated.

**Table 4:** Ultrasound accuracy in prospective and retrospective observational studies. CI: confidence interval; LR+: positive likelihood ratio; LR−: negative likelihood ratio

Study	Sensitivity % (95% CI)	Specificity % (95% CI)	LR+ (95% CI)	LR− (95% CI)	Accuracy % (95% CI)
Zanetta et al., 2003[17]	100 (29–100)	96 (86–99.5)	25 (6–95)	0	96 (87–99.5)
Bailleux et al., 2017 [4]	0	100 (63–100)	N/A	1 (1–1)	67 ( 35–90)
Pateman et al., 2014 [13]	N/A	100 (48–100)	N/A	N/A	N/A
Surampudi et al., 2015 [15]	N/A	N/A	N/A	N/A	N/A
Eichenberger-Gautschi et al., 2018 [13]	100 (2.5–100)	100 (1.59–100)	N/A	0	100 (63–100)
Mascilini et al., 2014 [16]	N/A	0 (0–34)	N/A	N/A	N/A
Dobashi et al., 2012 [18]	100 (69–100)	N/A	N/A	N/A	N/A

Of the 559 masses, 232 (42%) were excised: 115 antenatally, 77 at caesarean section, 36 post-partum, two following termination, and for two the timing was unknown. MRI was utilised for 31 masses; MRI findings agreed with ultrasound findings in 28/31 (90%) masses. Of the three cases of disagreement, MRI proved superior in two cases.

Pooled Results

Only studies with extractable 2 × 2 contingency tables were included in the final meta-analysis. Due to the high risk of bias and their relatively small weight, the study by Surampudi et al. [15], as well as all case reports and case series were excluded.

**Figure 2:**
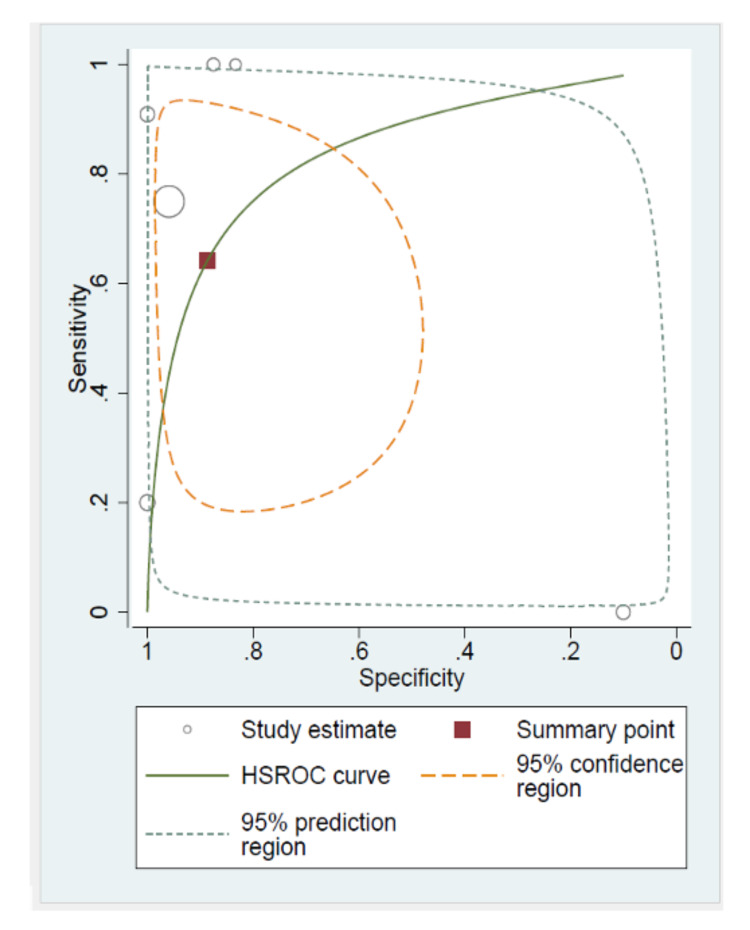
sROC curve for the diagnostic utility of ultrasound during pregnancy. sROC: summary receiver-operating characteristics

Overall, pooled sensitivity, specificity, LR+, and LR− of ultrasound for detecting ovarian malignancy were 64% (95% CI: 30-88%), 88% (95% CI: 64-97%), 5.6 (95% CI: 1.2-25.4), and 0.4 (95% CI: 0.15-1), respectively. We were unable to construct a Forest plot owing to the number of missing sensitivity or specificity values in some of the studies. Figure 2 illustrates the hierarchical sROC curve with the summary point in relation to the different study estimates.

This is the first systematic review to appraise the reliability of ultrasound in assessing adnexal masses in pregnancy. Our database search yield was large, which enabled an extensive review of the literature. Patient and study demographics were broad, allowing for the generalisability of the review.

Because only two studies used IOTA Simple Rules for ultrasound interpretation, it is presumed that others used pattern recognition (PR) [7,17]. PR is a subjective technique for assessing the morphological features of an adnexal mass on ultrasound and for predicting the nature of the mass and its histological type [93]. Several studies have demonstrated PR to be the most accurate means of assessing adnexal masses, with an accuracy rate of 92% [94]. The pooled accuracy rate in this review was 74% which is considerably lower. One reason for this may be that the original study by Timmerman et al. did not include adnexal masses in pregnant women [94]. Additionally, they showed that while results were comparable between specialist gynaecologists and sonographers, in less experienced hands, the accuracy levels decreased to 82% with a moderate interobserver agreement [94]. In this review, only one study reported that the ultrasound practitioner had more than 10 years of experience, which may explain the lower accuracy rate. While uncertainty over the level of experience of ultrasound practitioners may be seen as a limitation, it increases the generalisability of this review, as most antenatal ultrasounds are performed by practitioners with varying degrees of gynaecological experience.

Studies in non-pregnant women have shown that ultrasound can determine the nature of an adnexal mass in approximately 76% of cases [94]. In this review, 16 of the 559 (3%) masses were unclassifiable. There is no obvious reason for this small number. However, it may be due to publication bias, with clinicians less inclined to publish reports of undiagnosed cases. For unclassifiable masses, MRI has been suggested to be a useful adjunct. One study of 95 unclassifiable masses on ultrasound found MRI to have a 100% sensitivity for detecting malignancy and a 94% specificity for benign lesions, with excellent agreement between MRI and histological classification (k = 0.94) [95]. Of the seven masses unclassifiable on ultrasound in this review, six underwent MRI. Five were also unclassifiable on MRI, and the other was reported to be a dysgerminoma but was found to be a fibrothecoma on histology. In this review, MRI agreed with ultrasound diagnosis in 90% of cases, which is consistent with other studies [96]. While this review does not suggest an obvious superiority of MRI, it is a very small sample number. Moreover, all studies to date that have demonstrated the effectiveness of MRI in unclassifiable masses have been conducted among non-pregnant women using gadolinium. MRI in pregnancy requires specific protocols and a subspecialist set of skills that may not be widely available [97].

This review suggests that simple/functional cysts are the most common type of adnexal masses, which is consistent with previous studies [2]. However, the high prevalence of endometriomas compared to mature teratomas and other benign cysts was unexpected [98]. This is almost certainly due to two studies assessing endometriomas, with relatively large sample sizes. This review highlights the challenges endometriomas can cause in pregnancy. In the non-pregnant state, endometriomas can be diagnosed on ultrasound with a sensitivity and specificity of 92% and 97%, respectively [94]. However, in pregnancy, decidualisation occurs which may mimic malignancy on ultrasound. This was well represented in the study by Mascilini et al. in which all 18 endometriomas were diagnosed as a borderline ovarian tumour (BOT), malignant or unclassifiable on ultrasound [16]. Within case reports and case series, numerous presumed malignant masses were decidualised endometriomas on histology. MRI has been shown to be of benefit in such cases as the concerning areas of solid growth within a decidualised endometrioma show similar signal intensity as the decidualised endometrium [62]. This review supported these findings.

Because surgery during pregnancy carries greater risks to the mother and fetus, generally, the only indications to operate are torsion, haemorrhage, or suspicion of malignancy. In this review, 115 women underwent surgery during the antenatal period and 77 at the time of caesarean section. In the majority of cases, it was unclear if a caesarean section would have been performed for other indications. Of these 193 cases, 27 (14%) were malignant, eight (4%) were BOTs, and six (3%) had undergone torsion. This suggests that 152 (79%) cases may have undergone unnecessary surgery during pregnancy. This emphasises the need for high-quality research to determine the reliability of ultrasound in assessing adnexal masses in pregnancy, which could facilitate conservative management where appropriate and reduce maternal and fetal morbidity.

The low quality of the available evidence is the greatest limitation of this review. As expected, the vast majority of studies were case reports and case series, with only one prospective trial. Hence, good-quality prospective trials need to be conducted. The impact of this limitation was adjusted through the use of validated quality assessment tools and ranking the studies based on these tools. Due to the varied methodology adopted in retrospective observational studies, it was not possible to perform a complete statistical analysis involving all studies. Therefore, these studies are presented individually in the results. Despite not being able to perform a meta-analysis due to the heterogeneity of the studies with unacceptably wide CIs, pooled results offer a meaningful interpretation of the overall reliability of ultrasound.

## Conclusions

This review highlights the effectiveness of ultrasound in assessing adnexal masses in pregnancy. Due to a lack of strong evidence, it is still unclear if ultrasound is as reliable in pregnancy as it is in non-pregnant patients. Similarly, the accuracy of MRI is yet to be determined. Ratification of ultrasound models such as IOTA Simple Rules in pregnancy as well as further training of ultrasound practitioners in this field will allow for more accurate counselling and informed decision-making. It should be remembered that malignancy is rare in this cohort, and, as such, conservative management should be the default treatment approach. Subjective impressions from experts in this field along with close follow-up in suspicious lesions can reduce unnecessary surgeries during pregnancy.

## Appendices

**Table 5:** Search strategy for PubMed January 2021.

Search	Query	Results
#5	#1 AND #2 AND #3 AND #4	1,089
#4	“pregnant women”[MeSH Terms] OR “pregnancy”[Title/Abstract] OR “pregnant”[Title/Abstract] OR “gravid”[Title/Abstract] OR “antenatal”[Title/Abstract] OR “gestational”[Title/Abstract]	309,769
#3	“adnexa uteri”[MeSH Terms] OR “ovary”[Title/Abstract] OR “ovarian”[Title/Abstract] OR “fallopian tube”[Title/Abstract] OR “tubal”[Title/Abstract] OR “broad ligament”[Title/Abstract] OR “parametrial”[Title/Abstract] OR “parametrium”[Title/Abstract]	154,002
#2	“ultrasonography”[MeSH Terms] OR “imaging”[Title/Abstract] OR “ultrasound”[Title/Abstract] OR “sonography”[Title/Abstract] OR “sonogram”[Title/Abstract] OR “transvaginal”[Title/Abstract] OR “trans-abdominal”[Title/Abstract] OR “computer tomography”[Title/Abstract] OR “CT”[Title/Abstract] OR “magnetic resonance”[Title/Abstract] OR “MR”[Title/Abstract] OR “MRI”[Title/Abstract]	1,255,107
#1	“ovarian cysts”[MeSH Terms] OR “mass”[Title/Abstract] OR “tumour”[Title/Abstract] OR “tumor”[Title/Abstract] OR “neoplasm”[Title/Abstract] OR “malignancy”[Title/Abstract] OR “borderline”[Title/Abstract] OR “adenoma”[Title/Abstract] OR “dermoid”[Title/Abstract] OR “teratoma”[Title/Abstract] OR “corpus”[Title/Abstract] OR “corpora”[Title/Abstract] OR “endometrioma”[Title/Abstract] OR “cyst”[Title/Abstract]	1,768,737

**Table 6:** Search strategy for EMBASE January 2021.

Search	Query	Results
#5	#1 AND #2 AND #3 AND #4	3,824
#4	exp pregnancy/ or pregnancy.mp OR exp pregnant woman OR gravid.mp OR antenatal.mp OR gestational.mp	1,424,413
#3	ultrasound.mp. or exp ultrasound OR Transvaginal.mp OR transabdominal.mp OR computer assisted tomography.mp. or exp OR CT.mp OR magnetic resonance.mp OR MRI.mp	4,745,320
#2	mass.mp. or exp mass OR exp cyst/ or cyst.mp. or exp ovary cyst OR tumour.mp. or exp neoplasm OR malignant.mp OR exp adenoma/ or adenoma.mp OR dermoid.mp. or exp teratoma OR endometrioma.mp OR corpus.mp. or exp corpus luteum	8,230,355
#1	exp uterine adnexa OR adnexa*.mp. OR exp ovary OR ovarian.mp. OR fallopian.mp. or exp Fallopian tube OR broad ligament.mp. or exp broad ligament OR exp parametrium OR parametrial.mp	697,802

**Table 7:** STROBE checklist for risk of bias assessment. STROBE: Strengthening the Reporting of Observational Studies in Epidemiology

Description of items	First author (year)
1a: Study design is clear in title or abstract	0/1/NA
1b: Abstract provides an informative and balanced summary	
2: Explain the scientific background and rationale for the investigation being reported	
3: State specific objectives, including any prespecified hypotheses	
4: Present key elements of study design early in the paper	
5: Describe the setting, locations, and relevant dates, including periods of recruitment, exposure, follow-up, and data collection	
6a: Give the eligibility criteria and the sources and methods of selection of participants; describe methods of follow-up	
6b: For matched studies, give matching criteria and number of exposed and unexposed	
7: Clearly define all outcomes, exposures, predictors, potential confounders, and effect modifiers; give diagnostic criteria, if applicable	
8: For each variable of interest, give data sources and details of methods of assessment	
9: Describe any efforts to address potential sources of bias	
10: Explain how the study size was arrived at	
11: Explain how quantitative variables were handled in the analyses; if applicable, describe which groupings were chosen and why	
12a: Describe all statistical methods	
12b: Describe any methods used to examine subgroups and interactions	
12c: Explain how missing data were addressed	
12d: Explain how loss to follow-up was addressed	
12e: Describe any sensitivity analyses	
13a: Report numbers of individuals at each stage of study	
13b: Give reasons for non-participation at each stage	
13c: Consider use of a flow diagram	
14a: Give characteristics of study participants and information on exposures and potential confounders	
14b: Indicate number of participants with missing data for each variable of interest	
14c: Summarize follow-up time	
15: Report numbers of outcome events or summary measures over time	
16a: Give unadjusted estimates and, if applicable, confounder-adjusted estimates and their precision	
16b: Report category boundaries when continuous variables were categorized	
16c: If relevant, consider translating estimates of relative risk into absolute risk for a meaningful time period	
17: Report other analyses done	
18: Summarize key results with reference to study objectives	
19: Discuss limitations of the study, taking into account sources of potential bias or imprecision.	
20: Give a cautious overall interpretation of results	
21: Discuss the generalisability of the study results	
22: Give funding sources	
Summary	X/Y (%)

**Table 8:** Joanna Briggs Institute checklist: case reports.

	Yes (2)	No (0)	Unclear (1)	N/A	0/1/2
Were patient’s demographic characteristics clearly described?	□	□	□	□	
Was the patient’s history clearly described and presented as a timeline?	□	□	□	□	
Was the current clinical condition of the patient on presentation clearly described?	□	□	□	□	
Were diagnostic tests or assessment methods and the results clearly described?	□	□	□	□	
Was the intervention(s) or treatment procedure(s) clearly described?	□	□	□	□	
Was the post-intervention clinical condition clearly described?	□	□	□	□	
Were adverse events (harms) or unanticipated events identified and described?	□	□	□	□	
Does the case report provide takeaway lessons?	□	□	□	□	
Total					X/Y %

**Table 9:** Joanna Briggs Institute checklist: case series.

	Yes (2)	No (0)	Unclear (1)	N/A	0/1/2
Were there clear criteria for inclusion in the case series?	□	□	□	□	
Was the condition measured in a standard, reliable way for all participants included in the case series?	□	□	□	□	
Were valid methods used for identification of the condition for all participants included in the case series?	□	□	□	□	
Did the case series have consecutive inclusion of participants?	□	□	□	□	
Did the case series have complete inclusion of participants?	□	□	□	□	
Was there clear reporting of the demographics of the participants in the study?	□	□	□	□	
Was there clear reporting of clinical information of the participants?	□	□	□	□	
Were the outcomes or follow-up results of cases clearly reported?	□	□	□	□	
Was there clear reporting of the presenting site(s)/clinic(s) demographic information?	□	□	□	□	
Was statistical analysis appropriate?	□	□	□	□	
Total					X/Y %
